# Planar Hypercoordinate FeB_3_ Monolayer for Universal Alkali-Ion Storage with Ultrahigh Capacity and Ultralow Diffusion Barriers

**DOI:** 10.3390/ma19214493

**Published:** 2026-10-25

**Authors:** Huanhuan Xie, Kehan Zhang, Dong Liang, Pinpin Jing, Changding Guo

**Affiliations:** 1 Key Laboratory of Magnetic Molecules and Magnetic Information Materials of the Ministry of Education, School of Materials Science and Engineering, Shanxi Normal University, Taiyuan 030031, China; 225111085@sxnu.edu.cn (K.Z.); 226125059@sxnu.edu.cn (D.L.); 226125036@sxnu.edu.cn (P.J.); 2 Center for Integrated Nanostructure Physics (CINAP), Sungkyunkwan University, Suwon 16419, Republic of Korea; 3 Department of Energy Science, Sungkyunkwan University, Suwon 16419, Republic of Korea; cd.guo@skku.edu

**Keywords:** FeB_3_ monolayer, alkali-metal-ion batteries, universal anode, ultralow diffusion barrier, first-principles calculations

## Abstract

**Highlights:**

- One planar FeB_3_ host supports lithium, sodium, and potassium storage.
- B–B–B hollow sites are preferred by all three alkali-metal species.
- Migration barriers are 0.041, 0.008, and 0.025 eV for lithium, sodium, and potassium.
- Theoretical capacities reach 944.2, 809.3, and 606.9 mAh g^−1^, respectively.
- The Fe–B framework remains intact under high alkali-metal loading.

**Abstract:**

Developing a single anode host capable of high-capacity and fast storage of Li, Na, and K remains a major challenge for next-generation alkali-metal-ion batteries. Herein, the electrochemical performance of a planar hypercoordinate FeB_3_ monolayer is systematically investigated using first-principles calculations. The FeB_3_ monolayer features a metallic electronic structure and an exposed boron-rich surface, providing favorable conditions for alkali-metal adsorption. Li, Na, and K preferentially occupy the B–B–B hollow regions and undergo substantial charge transfer to the substrate. The calculated migration barriers are only 0.041, 0.008, and 0.025 eV for Li, Na, and K, respectively, indicating ultrafast surface diffusion. Open-circuit-voltage analysis gives accessible theoretical capacities of 944.2, 809.3, and 606.9 mAh g^−1^ for Li-, Na-, and K-ion storage, respectively. Moreover, the Fe–B framework remains largely intact under high alkali-metal loading. These results identify planar FeB_3_ as a promising universal anode combining metallicity, ultrafast ion transport, high capacity, and structural compatibility.

## 1. Introduction

The rapid growth of portable electronics, electric vehicles, and large-scale grid storage has created an urgent demand for rechargeable batteries with higher energy density, faster charging capability, longer cycle life, and improved resource sustainability [1,2,3]. Lithium-ion batteries (LIBs) have dominated the current energy-storage market because of their high operating voltage and mature technological development [4,5,6]. However, the uneven distribution and limited abundance of lithium resources, together with increasing cost and safety concerns associated with high-rate operation, have stimulated intensive efforts to explore alternative alkali-metal-ion battery systems. Sodium-ion batteries (SIBs) and potassium-ion batteries (PIBs), benefiting from the natural abundance and low cost of Na and K, are regarded as promising complements or alternatives to LIBs, particularly for large-scale stationary energy storage [7,8,9]. Despite these advantages, the development of high-performance anode materials remains a critical bottleneck for alkali-metal-ion batteries, especially because Na^+^ and K^+^ possess larger ionic radii and slower diffusion kinetics than Li^+^.

Graphite, the commercial anode for LIBs, has a limited theoretical capacity of 372 mAh g^−1^ and shows unsatisfactory compatibility with Na-ion storage. Meanwhile, many alloying- and conversion-type anodes can deliver high capacities but usually suffer from sluggish ion transport, large volume variation, structural degradation, or poor rate capability during repeated cycling [10,11]. More importantly, although Li-, Na-, and K-ion batteries share similar working principles, a host material that performs well for one alkali ion does not necessarily exhibit favorable electrochemical behavior for the others [12]. Therefore, identifying a universal anode host that can simultaneously accommodate Li, Na, and K ions with high capacity, low diffusion barriers, suitable voltage, and structural robustness is highly desirable but remains challenging.

Two-dimensional materials provide an attractive platform for designing advanced battery anodes because of their large specific surface area, abundant exposed active sites, short ion-diffusion pathways, and tunable electronic structures [13,14,15]. Representative 2D materials, including graphene derivatives, phosphorene, transition-metal dichalcogenides, MXenes, MBenes, and boron-based monolayers, have been widely investigated as anode candidates for metal-ion batteries [16,17,18,19]. Among them, boron-rich two-dimensional materials are particularly appealing [20,21]. The electron-deficient nature of boron enables multicenter bonding, flexible coordination environments, and strong interactions with metal adatoms, which can facilitate stable alkali-metal adsorption and fast surface diffusion [22,23,24]. However, pristine boron-based sheets often face challenges such as structural polymorphism, anisotropic stability, or insufficient electronic/chemical optimization. Introducing transition-metal atoms into boron frameworks offers an effective route to modulate the electronic structure, stabilize planar networks, and create highly active adsorption sites.

Iron borides represent an emerging family of two-dimensional metalloborophenes with unusual bonding configurations and rich electronic properties. Previous first-principles calculations and global structure searches have identified diverse low-dimensional Fe–B structures, ranging from multilayer sandwich-like phases to planar monolayers. In particular, a planar hypercoordinate FeB_3_ monolayer has been theoretically predicted, in which Fe atoms are incorporated into a boron framework, giving rise to high structural stability, metallic conductivity, and intriguing magnetoelectronic properties. These structural features are also highly relevant to electrochemical energy storage: the planar Fe–B network can provide continuous electron transport channels, while the boron-rich surface and multiple coordination sites may enable strong yet reversible alkali-metal adsorption. Nevertheless, the potential of planar FeB_3_ as a universal anode material for Li-, Na-, and K-ion batteries has not been systematically clarified.

Herein, we systematically investigate the electrochemical performance of a planar FeB_3_ monolayer as a universal anode for LIBs, SIBs, and PIBs by first-principles calculations. The intrinsic structural stability, electronic conductivity, alkali-metal adsorption behavior, charge-transfer characteristics, ion-diffusion kinetics, theoretical storage capacity, open-circuit voltage, and adsorption-induced structural stability are comprehensively evaluated. Our results reveal that FeB_3_ combines several essential merits required for high-performance anodes: intrinsic metallic conductivity, strong adsorption toward Li/Na/K atoms, ultralow diffusion barriers, high theoretical capacity, and robust structural integrity upon alkali-metal loading. These findings suggest that planar FeB_3_ is a promising universal anode candidate for high-rate alkali-metal-ion batteries and provide useful insights into the design of boron-rich metalloborophene electrodes for next-generation energy-storage applications.

## 2. Computational Methods

All first-principles calculations were performed within the framework of density functional theory (DFT) using the DS-PAW package implemented in Device Studio [25]. The projector augmented wave (PAW) method was adopted to describe the electron–ion interactions [26], and the Perdew–Burke–Ernzerhof (PBE) functional within the generalized gradient approximation (GGA) was used for the exchange–correlation potential [27]. Long-range van der Waals interactions were considered using Grimme’s DFT-D3 correction [28]. A vacuum layer of at least 15 Å was introduced perpendicular to the monolayer to avoid artificial interactions between periodically repeated images. For the primitive FeB_3_ cell, a dense Monkhorst–Pack k-point mesh of 15 × 15 × 1 was used for structural optimization and electronic-structure calculations. For adsorption, diffusion, and high-coverage storage calculations, a 3 × 3 × 1 FeB_3_ supercell was employed, together with a 3 × 3 × 1 k-point mesh. The plane-wave cutoff energy was set to 550 eV. The convergence criteria for structural relaxation were set to 10^−4^ eV for the total energy and 0.01 eV Å^−1^ for the residual force on each atom. The migration energy barriers were calculated using the climbing-image nudged elastic band (CI-NEB) method [29]. The migration energy barriers were determined from the maximum energy along the converged minimum-energy pathway relative to the initial state.

During the preparation of the graphical abstract, the authors used ChatGPT Images 2.5 (OpenAI, San Francisco, CA, USA) to generate selected schematic illustrations. The authors have reviewed and edited the output and take full responsibility for the content of this publication.

## 3. Results and Discussion

### 3.1. Crystal Structure and Electronic Properties of the FeB_3_ Monolayer

**Figure 1:**
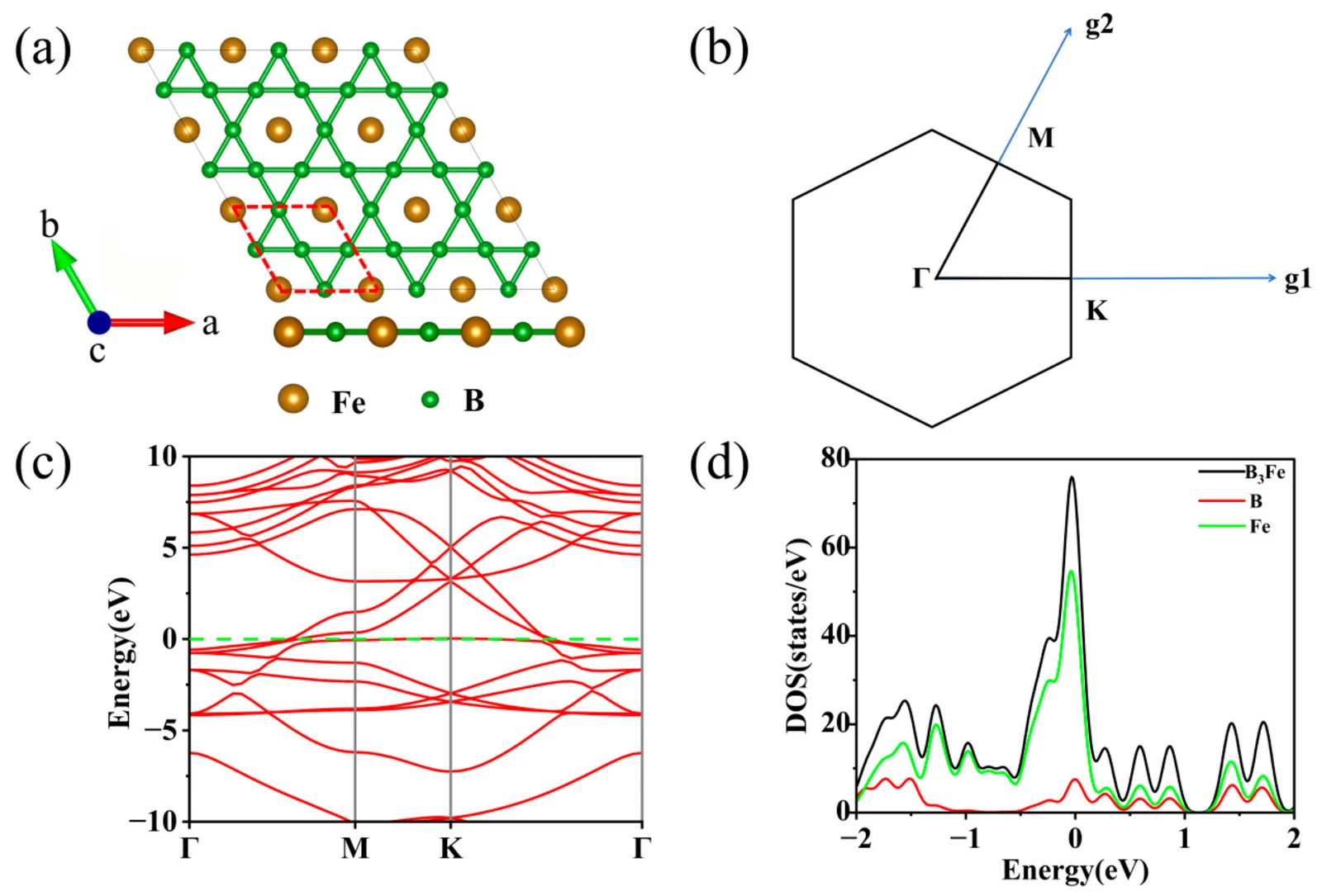
(**a**) Top and side views of the FeB_3_ monolayer. Brown and green spheres represent Fe and B atoms, respectively. The red dashed parallelogram marks the primitive cell. (**b**) Brillouin zone of the FeB_3_ monolayer. (**c**) Electronic band structure of the FeB_3_ monolayer. (**d**) Total and projected density of states of the FeB_3_ monolayer. The Fermi level is set to 0 eV.

The previously reported β-FeB_3_ monolayer was adopted as the initial structure and fully relaxed before evaluating its alkali-metal-ion-storage properties. As shown in Figure 1a, the optimized monolayer retains a fully planar hexagonal configuration (space group P6/mmm). The B atoms form a kagome-like framework, whereas the Fe atoms constitute a trigonal sublattice and occupy the centers of the B hexagons. Each Fe atom is coordinated by six coplanar B atoms, characterizing this structure as a planar hexacoordinate metalloborophene. The primitive cell, highlighted by the red dashed parallelogram, contains one Fe atom and three B atoms. Such an exposed and atomically flat framework provides multiple accessible sites for subsequent alkali-metal adsorption.

In the present work, non-spin-polarized calculations were employed to investigate the electrochemical properties of the alkali-metal-loaded systems. This treatment represents an approximation and does not establish the magnetic ground state of either pristine or alkali-metal-loaded β-FeB_3_. In particular, magnetic reorganization induced by alkali-metal adsorption has not been explicitly examined. Accordingly, the calculated electrochemical properties should be interpreted within the adopted non-spin-polarized approximation. Figure 1b depicts the corresponding Brillouin zone and the high-symmetry k-point path used for the band-structure calculations. As shown in Figure 1c, several bands cross the Fermi level along the Γ–M–K–Γ path, demonstrating that the FeB_3_ monolayer exhibits a metallic electronic structure. This metallic character is further corroborated by the density of states in Figure 1d, which shows a finite DOS at the Fermi level. The projected density of states further reveals that both Fe- and B-derived states contribute to the electronic states near the Fermi level, with the Fe states providing the dominant contribution. The substantial overlap between the Fe and B states over the same energy range suggests appreciable Fe–B orbital hybridization. Taken together, the finite DOS at the Fermi level and the joint contributions from Fe and B states indicate favorable electronic conductivity, which is beneficial for electron transport during alkali-metal adsorption and electrochemical cycling.

To further elucidate the bonding characteristics of the Fe–B framework, the ELF map of the pristine FeB_3_ monolayer was analyzed, as shown in Figure S1b. Electron localization is predominantly associated with the B-containing framework, whereas regions with ELF values close to 0.5 display electron-gas-like delocalized character. The coexistence of localized and delocalized electronic features is therefore consistent with both the bonding characteristics and the metallic electronic structure of the planar Fe–B framework. Overall, the FeB_3_ monolayer combines an accessible planar architecture with metallic electronic states, providing favorable structural and electronic characteristics for its further evaluation as an anode material for alkali-metal-ion batteries.

### 3.2. Adsorption of Single Li, Na, and K Atoms on the FeB_3_ Monolayer

The adsorption behavior of single Li, Na, and K atoms on the FeB_3_ monolayer was first investigated using a 3 × 3 FeB_3_ supercell to minimize the interaction between periodically repeated adsorbates. As shown in Figure S2, six representative adsorption sites were considered, including the top site above a B atom (S1), the top site above an Fe atom (S2), the bridge site above a B–B bond (S3), the bridge site above a B–Fe bond (S4), the hollow site surrounded by three B atoms (S5), and the hollow site surrounded by B–B–Fe atoms (S6). The adsorption energy (*E*_ad_) was calculated according to the following equation [30]:

$$E_{\mathrm{ad}}=E_{M+{\mathrm{FeB}}_{3}}-E_{{\mathrm{FeB}}_{3}}-E_{M}$$

where $E_{M+{\mathrm{FeB}}_{3}}$ and $E_{{\mathrm{FeB}}_{3}}$ are the total energies of the FeB_3_ supercell with and without an adsorbed alkali-metal atom, respectively, and *E*_M_ is the energy per atom of bulk Li, Na, or K metal. A negative adsorption energy indicates that adsorption on the FeB_3_ monolayer is thermodynamically favorable with respect to metal aggregation, while a more negative value corresponds to stronger binding between the adsorbate and the substrate.

**Figure 2:**
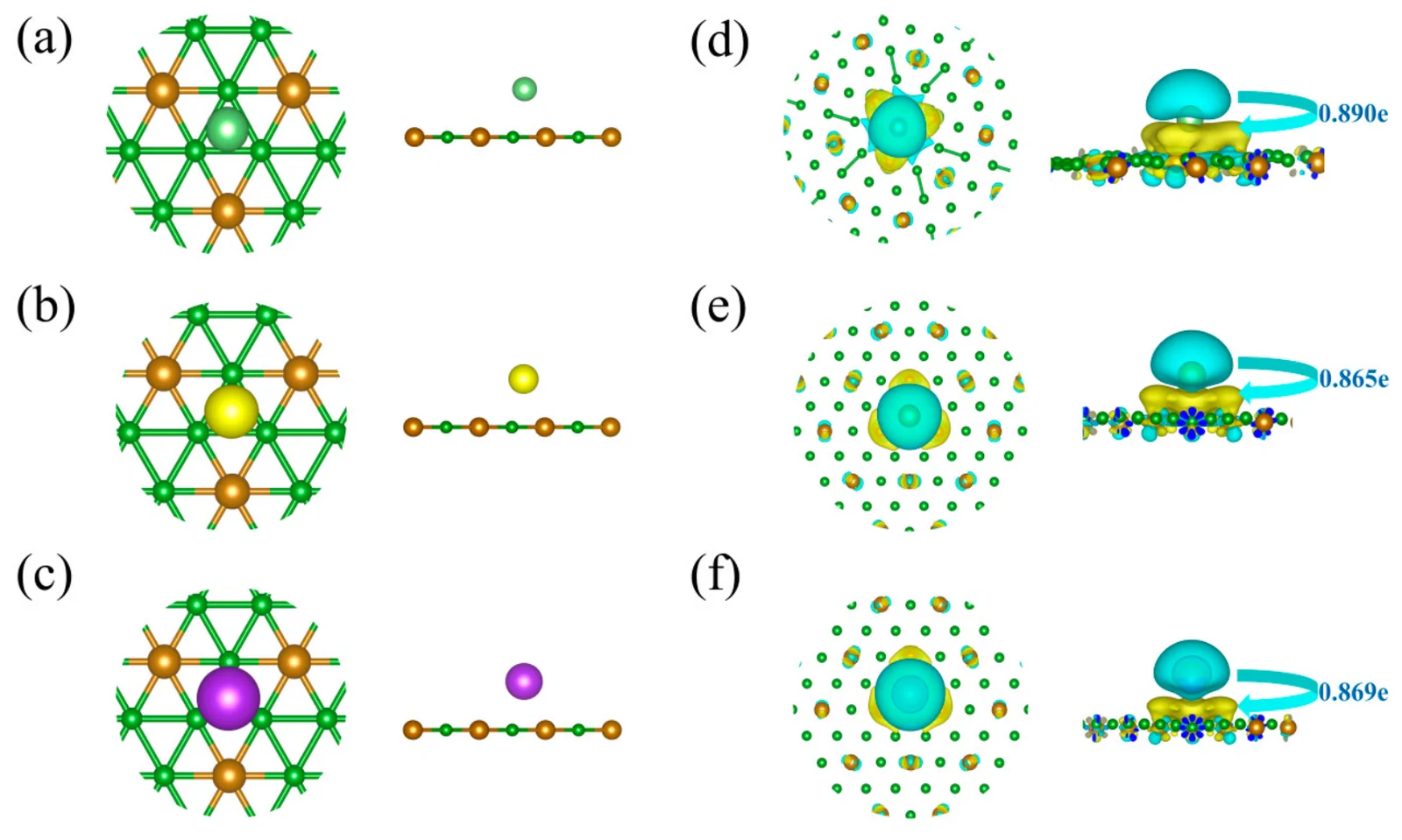
Optimized adsorption configurations and charge-density difference plots of Li, Na, and K atoms on the FeB_3_ monolayer. (**a**–**c**) The most stable adsorption configurations of Li, Na, and K atoms at the B–B–B hollow site, respectively. For each configuration, the left and right panels show the top and side views, respectively. (**d**–**f**) Charge-density difference plots of the Li-, Na-, and K-adsorbed FeB_3_ systems, respectively. The isosurface value is 0.01 e Å^−3^.

The calculated adsorption energies for all examined sites are summarized in Table S1. For Li adsorption, the B–B–B hollow site (S5) has the lowest calculated adsorption energy of −1.68 eV, followed closely by the B–B bridge site (S3, −1.66 eV). For Na adsorption, the S5 site also has the lowest calculated adsorption energy of −1.79 eV, only 0.01 eV lower than that of the B–B bridge site (S3, −1.78 eV). Similarly, the S5 site has a slightly lower calculated adsorption energy of −2.30 eV for K adsorption than the B–B bridge site (S3, −2.29 eV). These small energy differences indicate that the S3 and S5 sites are nearly degenerate, with S5 exhibiting only a slight energetic preference under the adopted computational settings. The optimized adsorption configurations of Li, Na, and K at the S5 site are shown in Figure 2a–c. The strong adsorption energies suggest robust interactions between alkali-metal atoms and the FeB_3_ monolayer, which may help suppress metal aggregation on the anode surface.

To further reveal the interaction between the adsorbed alkali-metal atoms and the FeB_3_ substrate, charge-density difference plots were analyzed, as shown in Figure 2d–f. The charge redistribution around the adsorbed atoms indicates pronounced electron transfer from Li, Na, and K to the FeB_3_ monolayer. This is further confirmed by Bader charge analysis, which shows that Li, Na, and K donate approximately 0.890, 0.865, and 0.869 *e*, respectively, to the FeB_3_ substrate. Therefore, the adsorbed alkali-metal atoms mainly exist in a cationic state on the FeB_3_ surface. The strong charge transfer and favorable adsorption energies demonstrate that the FeB_3_ monolayer can effectively anchor Li, Na, and K atoms, providing a stable basis for subsequent alkali-ion storage.

### 3.3. Diffusion of Li/Na/K Ions on the FeB_3_ Monolayer

**Figure 3:**
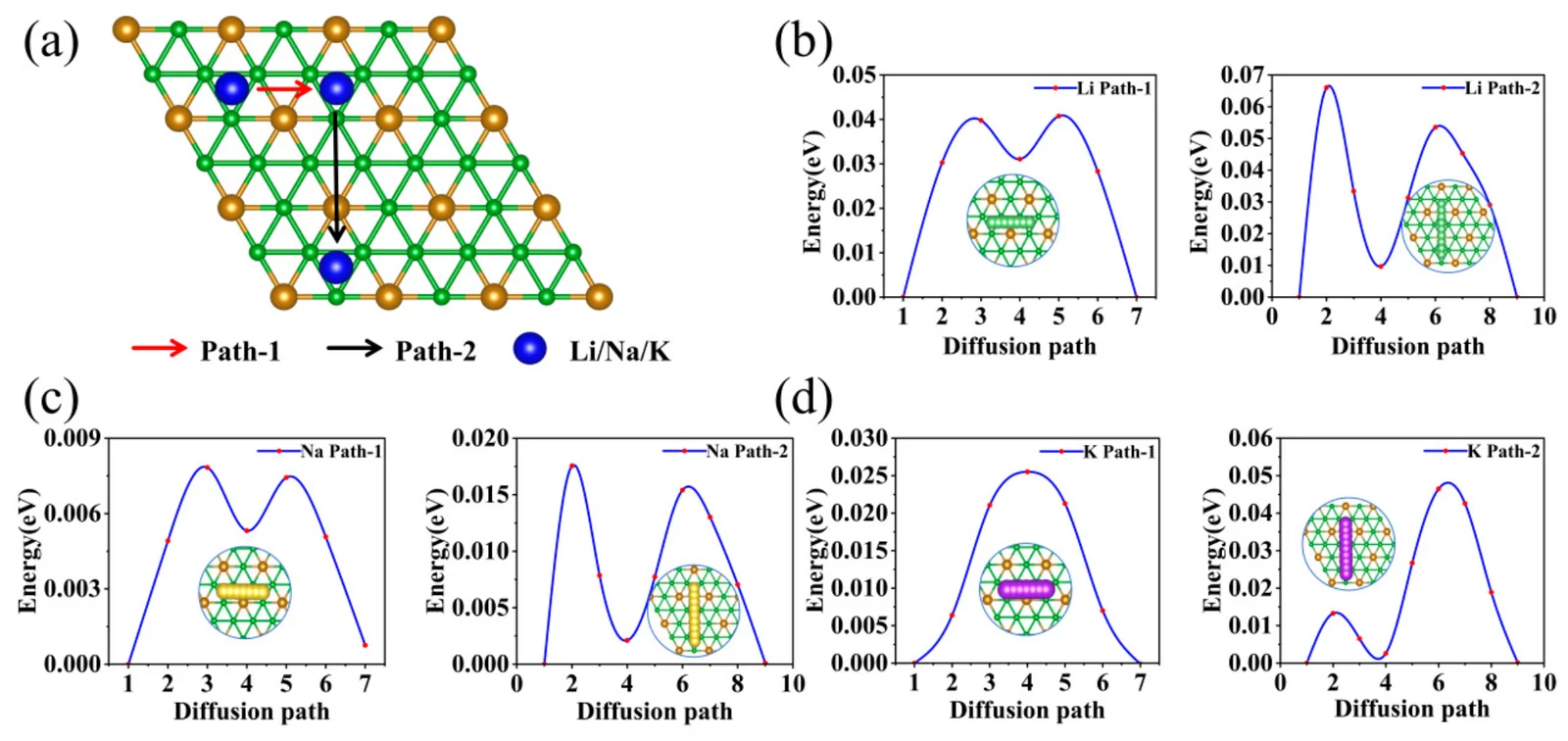
(**a**) Schematic illustration of two representative alkali-metal diffusion pathways on the FeB_3_ monolayer. The blue spheres denote Li/Na/K atoms at the most stable B–B–B hollow sites. (**b**–**d**) CI-NEB energy profiles for Li, Na, and K diffusion along Path-1 and Path-2, respectively.

Ion diffusion kinetics is a key factor governing the rate capability of battery anode materials. After identifying the most stable adsorption site, we further investigated the migration behavior of Li, Na, and K on the FeB_3_ monolayer using a 3 × 3 supercell. As shown in Figure 3a, two representative diffusion pathways connecting adjacent B–B–B hollow sites (S5) were considered and denoted as Path-1 and Path-2. The corresponding migration barriers were calculated using the CI-NEB method, as presented in Figure 3b–d. For Li, Na, and K migration along Path-1, the calculated diffusion barriers are only 0.041, 0.008, and 0.025 eV, respectively. The barriers along Path-2 are slightly higher, with values of 0.066 eV for Li, 0.017 eV for Na, and 0.046 eV for K. Therefore, Path-1 is energetically more favorable for all three alkali-metal species. Notably, Na exhibits the lowest migration barrier among the three ions, suggesting highly favorable Na-ion diffusion kinetics on the FeB_3_ surface. The ultralow barriers can be attributed to the open planar Fe–B framework and the smooth diffusion channels connecting neighboring hollow adsorption sites.

Compared with many representative two-dimensional anode materials, the diffusion barriers on FeB_3_ are remarkably low. For example, reported diffusion barriers are 0.063 eV for MoS_2_, 0.103 eV for Ti_3_C_2_, 0.11 eV for FeSe monolayer, 0.14 eV for SiGe, 0.155 eV for BP monolayer, 0.21 eV for θ-graphene, 0.26 eV for PC_6_, and 0.49 eV for MoN_2_ [31,32,33,34,35,36,37,38]. In contrast, the maximum barrier along the preferred Path-1 on FeB_3_ is only 0.041 eV, indicating much faster alkali-ion migration. These results suggest that the FeB_3_ monolayer possesses excellent ion-transport kinetics, which is expected to contribute to high-rate charge/discharge performance in Li-, Na-, and K-ion batteries. The reported diffusion barriers correspond to the alkali-metal species and adsorption conditions adopted in the respective studies.

### 3.4. Adsorption of Multiple Li/Na/K Ions on the FeB_3_ Monolayer Surface

The adsorption capacity of alkali-metal atoms is a key factor determining the theoretical storage capability of anode materials. After identifying the B–B–B hollow site (S5) as the most favorable adsorption site for single Li, Na, and K atoms, we further investigated the adsorption behavior of multiple alkali-metal atoms on both sides of the FeB_3_ monolayer. For each adsorption concentration, as many symmetry-distinct configurations as possible were considered within the 3 × 3 FeB_3_ supercell, including different spatial arrangements and distributions of alkali-metal atoms between the two surfaces. Asymmetric arrangements between the two surfaces were also considered. The B–B–B hollow site (S5) was used as the primary adsorption site based on the single-atom adsorption results in Section 3.2. For each configuration, the atomic positions were fully relaxed while the in-plane lattice vectors were kept consistent with those of the optimized pristine FeB_3_ monolayer, and the lowest-energy configuration among the examined arrangements was selected for further analysis.

Initially, single-layer adsorption on both sides of the FeB_3_ monolayer was considered. Full occupation of the available S5 sites corresponds to 36 adsorbed alkali-metal atoms in the 3 × 3 supercell. After structural relaxation, the average adsorption energy at n = 36 for Li remained negative, with a value of −0.55 eV. In contrast, for Na and K adsorption, the configurations containing 36 Na or K atoms gave positive adsorption energies, suggesting that such high Na/K loadings are energetically unfavorable. By gradually reducing the number of adsorbed Na and K atoms, the maximum loading with negative average adsorption energy was determined to be 28 atoms for Na and 18 atoms for K.

**Figure 4:**
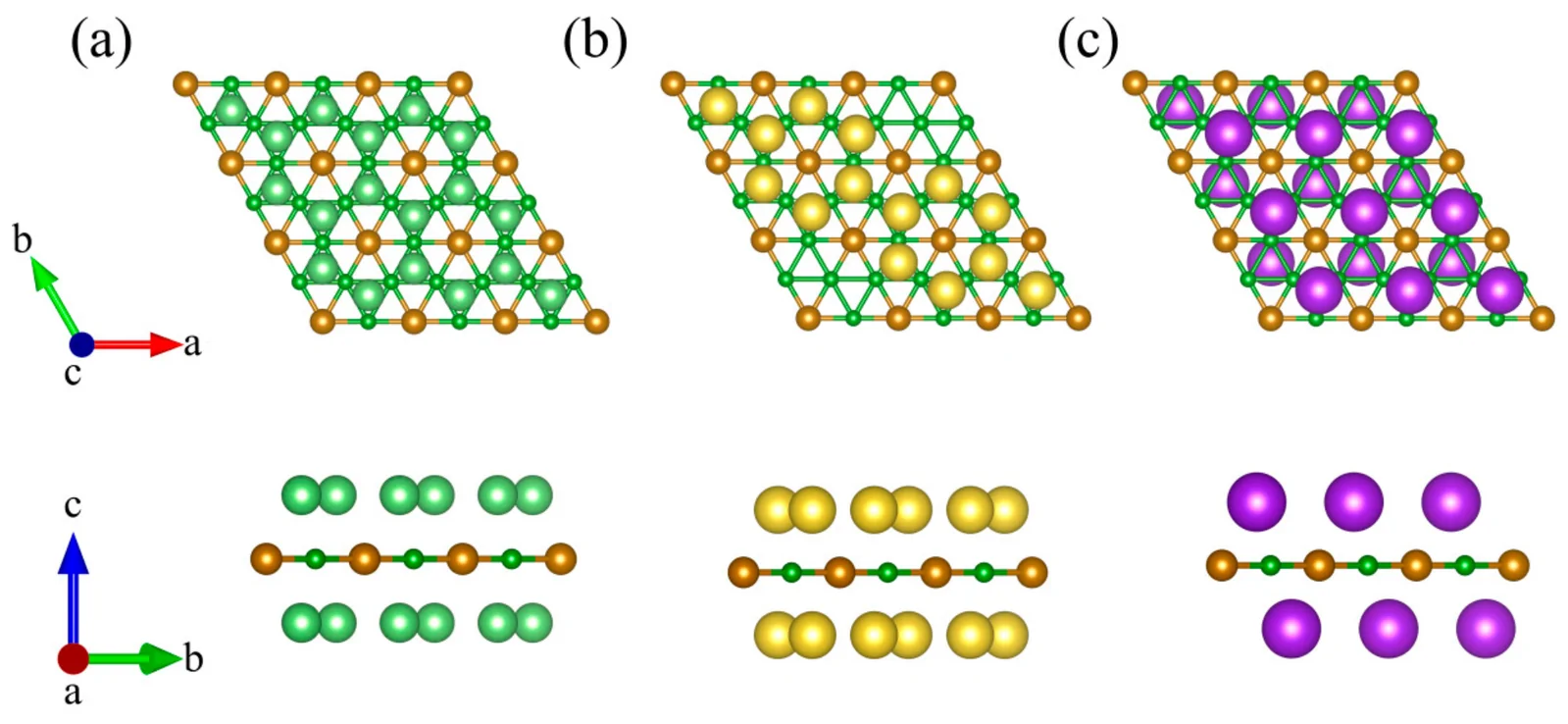
Optimized maximum-adsorption configurations of alkali-metal atoms on the FeB_3_ monolayer: (**a**) 36 Li atoms, (**b**) 28 Na atoms, and (**c**) 18 K atoms. For each configuration, the upper and lower panels show the top and side views, respectively.

**Figure 5:**
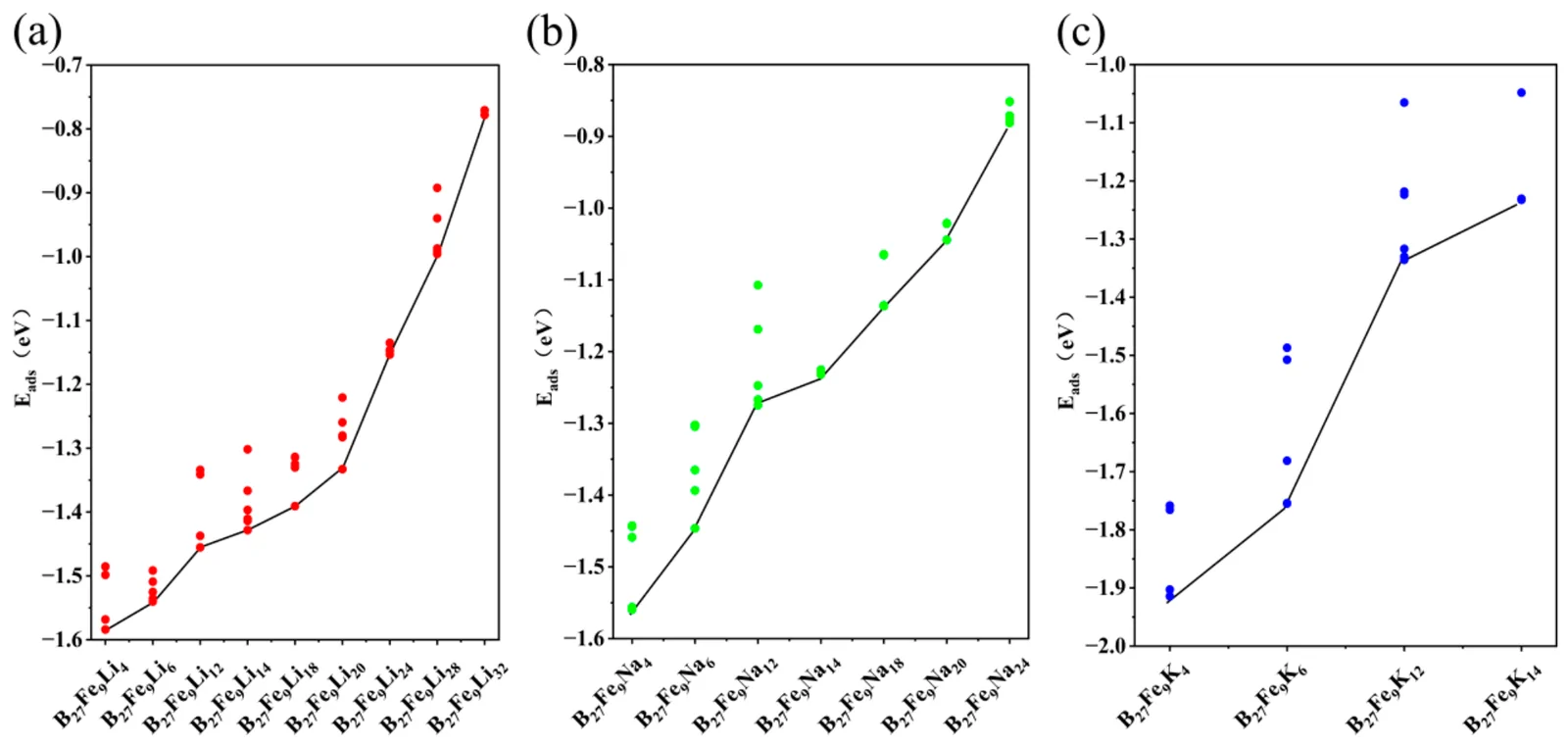
Average adsorption energy as a function of adsorption concentration for (**a**) Li, (**b**) Na, and (**c**) K atoms on the FeB_3_ monolayer.

The optimized maximum-adsorption configurations are shown in Figure 4. For Li, the maximum-adsorption configuration contains 36 Li atoms, corresponding to adsorption on both sides of the FeB_3_ monolayer. For Na and K, the maximum-loading configurations with negative average adsorption energies contain 28 and 18 atoms, respectively. To further clarify the adsorption evolution with increasing alkali-metal concentration, various intermediate adsorption configurations were examined, and the corresponding average adsorption energies are summarized in Table 1 and plotted in Figure 5. For Li adsorption, the average adsorption energy gradually increases from −1.58 eV at n = 4 to −0.55 eV at n = 36, but remains negative throughout the investigated concentration range. For Na adsorption, the average adsorption energy changes from −1.56 eV at n = 4 to −0.64 eV at n = 28. For K adsorption, the value increases from −1.91 eV at n = 4 to −0.97 eV at n=18. The gradual weakening of adsorption with increasing coverage can be attributed to enhanced repulsive interactions among adsorbed alkali-metal atoms at high concentrations.

Overall, the negative average adsorption energies indicate overall favorable adsorption relative to the corresponding bulk metals, with maximum adsorption numbers of 36 for Li, 28 for Na, and 18 for K in the 3 × 3 supercell. These high adsorption concentrations suggest that the FeB_3_ monolayer has considerable potential for high-capacity alkali-metal-ion storage. A negative average adsorption energy does not establish favorable incremental insertion; the storage limits are determined from the OCV profiles in the following section.

### 3.5. Open-Circuit Voltage and Theoretical Storage Capacity of the FeB_3_ Monolayer

**Figure 6:**
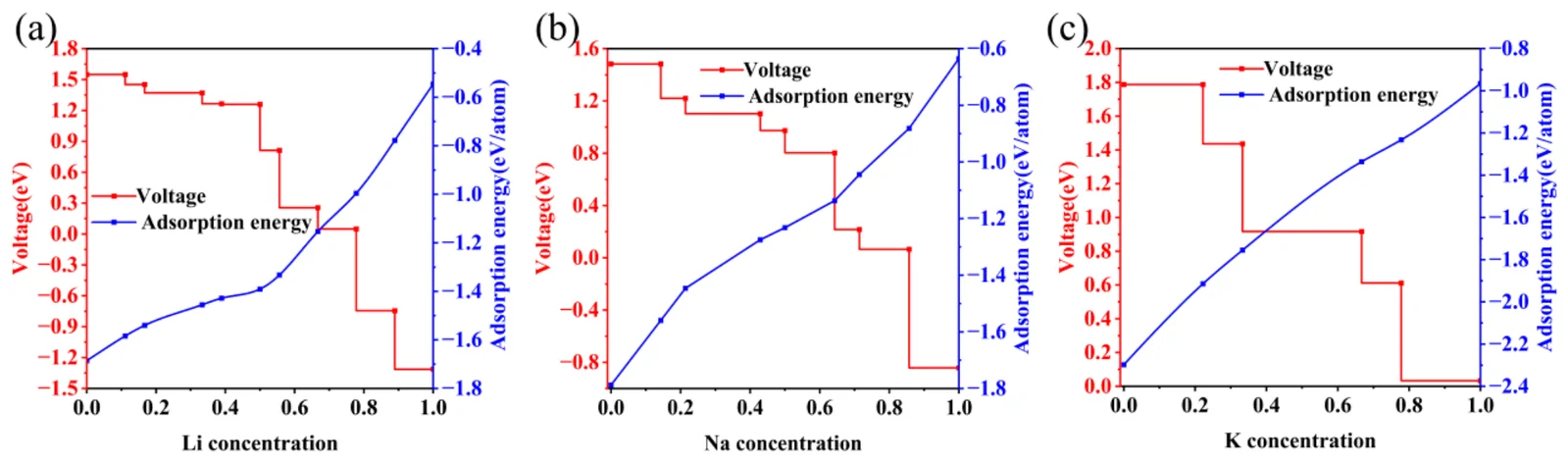
(**a**–**c**) Variations in the average adsorption energy and open-circuit voltage (OCV) of the FeB_3_ monolayer as a function of the concentration of adsorbed Li, Na, and K ions.

The average open-circuit voltage (OCV) over each successive loading interval reflects the incremental insertion thermodynamics relative to the corresponding bulk metal. For the FeB_3_ monolayer anode, the OCV is given by:

$$V\approx \frac{E_{(\mathrm{Li}/\mathrm{Na}/K)X_{1}}-E_{(\mathrm{Li}/\mathrm{Na}/K)X_{2}}+(X_{2}-X_{1})E_{\mathrm{Li}/\mathrm{Na}/K}}{X_{2}-X_{1}}$$

where 𝒳_1_ and 𝒳_2_ are the amounts of Li, Na, and K (𝒳_2_ > 𝒳_1_), and $E_{(\mathrm{Li}/\mathrm{Na}/K)X_{1}}$, $E_{(\mathrm{Li}/\mathrm{Na}/K)X_{2}}$ and E_Li/Na/K_ are the total energies of the two adsorption states and the per-atom energy of the corresponding bulk metal, respectively. The calculated OCV profiles are presented in Figure 6. As the alkali-metal concentration increases, the OCV decreases from 1.55 to −1.31 V for Li, from 1.48 to −0.84 V for Na, and from 1.79 to 0.03 V for K. Within the positive-OCV ranges, the mean voltages are 1.00 V (Li), 0.84 V (Na), and 0.96 V (K). These values represent theoretical anode potentials relative to the corresponding alkali-metal references, while the full-cell voltage additionally depends on the cathode potential. The last positive-voltage storage limits are 28 Li, 24 Na, and 18 K atoms per 3 × 3 supercell, respectively. Further Li or Na insertion gives negative incremental voltages, indicating that these higher coverages are not thermodynamically favorable relative to the corresponding bulk metals. A negative OCV does not by itself indicate structural instability. The positive-OCV loading fractions are therefore 77.8% (28/36) for Li and 85.7% (24/28) for Na, while the K loading fraction is 100%.

**Figure 7:**
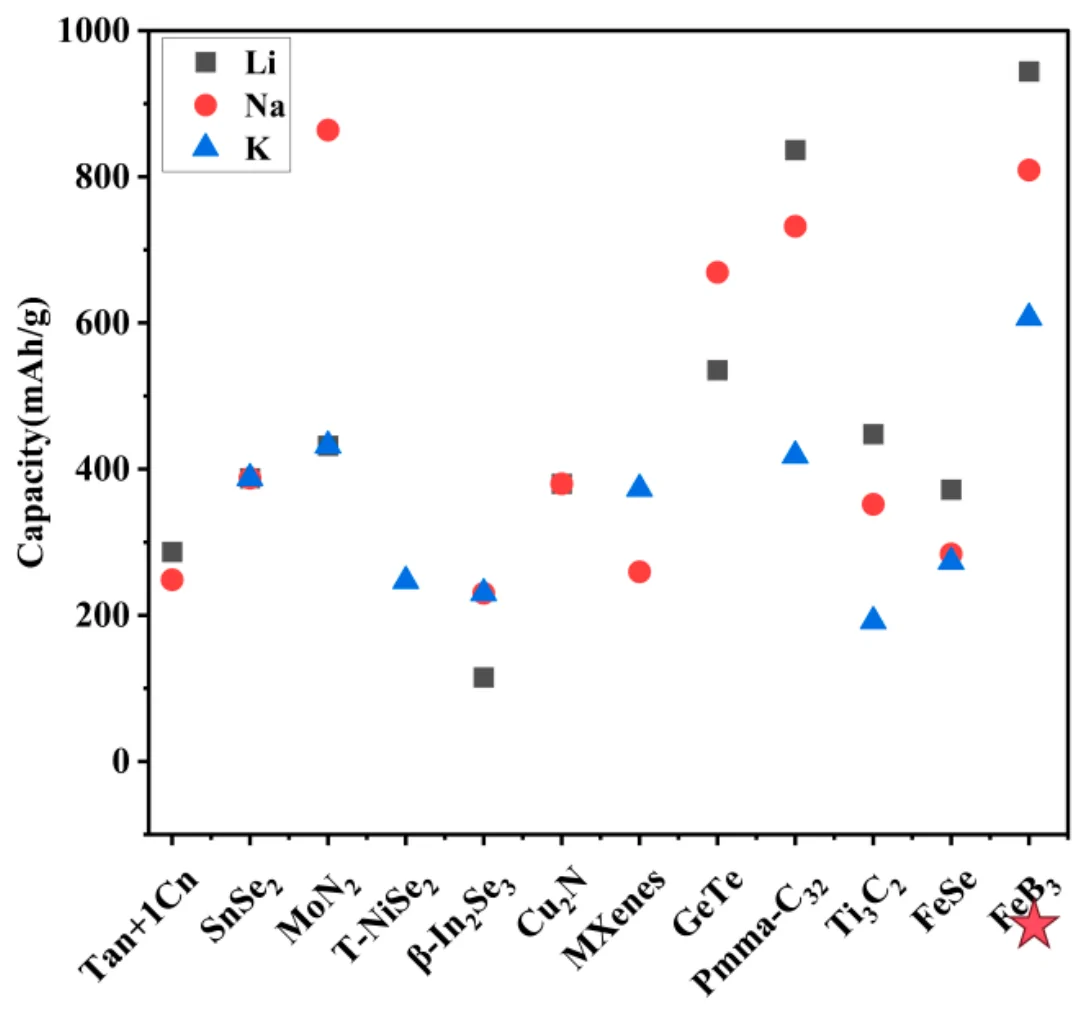
Comparison of the electrochemically accessible theoretical capacities of FeB_3_ with the reported capacities of representative 2D anode materials for Li-, Na-, and K-ion batteries. The red stars represent the results of the present work.

In addition to open-circuit voltage, high storage capacity constitutes another critical performance parameter for alkali-metal-ion batteries and serves as a principal benchmark for screening novel electrode materials. For these systems, the achievable capacity is fundamentally governed by the number of ions accommodated within the anode host: a higher ion loading directly translates into a greater theoretical capacity. The theoretical specific capacity is derived from the following equation [39]:

$$C=\frac{\omega F}{M_{\mathrm{FeB}3}}$$

where ω is the number of adsorbed ions per formula unit, F is the Faraday constant (26.8 Ah mol^−1^), and M_FeB3_ is the molar mass of FeB_3_. The maximum loadings with negative average adsorption energies of 36 Li, 28 Na, and 18 K atoms correspond to ideal adsorption-based capacities of approximately 1214.0, 944.2, and 606.9 mAh g^−1^, respectively. However, using the last positive-OCV loading limits of 28 Li, 24 Na, and 18 K atoms gives electrochemically accessible theoretical capacities of 944.2, 809.3, and 606.9 mAh g^−1^, respectively, as summarized in Table 2. These values are compared with those of representative two-dimensional anode materials [36,37,38,40,41,42,43,44,45,46,47] as shown in Figure 7. The positive-OCV cutoff, rather than the largest loading with negative average adsorption energy alone, determines the reported storage capacities.

### 3.6. Structural Integrity of the FeB_3_ Monolayer Under High Alkali-Metal Loading

Preservation of the host framework under high alkali-metal loading is essential for high-capacity anodes because excessive adsorption may induce buckling, bond breaking, or surface reconstruction. We therefore examined the optimized geometries and electron localization function (ELF) distributions of the pristine and Li-, Na-, and K-loaded FeB_3_ systems.

As shown in Figure S1a,c,e,g, the Fe–B framework remains nearly planar after structural relaxation at the representative high loadings of 36 Li, 28 Na, and 18 K atoms in the 3 × 3 supercell. No obvious bond breaking, severe buckling, or collapse of the Fe–B skeleton is observed, indicating that the FeB_3_ monolayer can accommodate substantial alkali-metal loading while retaining its underlying framework.

The corresponding ELF maps in Figure S1b,d,f,h provide further insight into electronic localization upon adsorption. In the pristine monolayer, localized features within the B-containing framework coexist with more delocalized regions in the planar sheet. After Li, Na, or K adsorption, the characteristic localization pattern associated with the Fe–B and B–B framework is largely preserved, suggesting that the substrate bonding network is not substantially disrupted at high coverage. Changes in ELF around the adsorbates and interfacial regions reflect modified electronic localization upon adsorption. Together with the charge-density-difference and Bader charge analyses discussed above, these features are consistent with appreciable electronic interactions between the alkali-metal atoms and the FeB_3_ substrate.

Taken together, the optimized high-loading structures and ELF distributions show that the FeB_3_ monolayer retains its nearly planar framework and essential bonding characteristics after Li, Na, and K adsorption. These results demonstrate structural preservation after relaxation and support the structural compatibility of FeB_3_ with high alkali-metal loading.

## 4. Conclusions

This study demonstrates that planar FeB_3_ is a promising host for Li-, Na-, and K-ion storage by combining favorable adsorption thermodynamics, rapid surface diffusion, and high storage capacity. Its exposed boron-rich framework provides accessible adsorption sites for all three alkali metals, while the metallic electronic structure supports charge transport. Importantly, FeB_3_ achieves strong alkali-metal binding without sacrificing ion mobility, as evidenced by the ultralow diffusion barriers of 0.041, 0.008, and 0.025 eV for Li, Na, and K, respectively. The storage behavior exhibits clear ion-dependent characteristics. Li reaches the highest electrochemically accessible theoretical capacity of 944.2 mAh g^−1^, whereas the electrochemically accessible capacities for Na and K are 809.3 and 606.9 mAh g^−1^, respectively. For Na, the negative voltage of the final insertion step shows that the practical storage limit should be determined from the voltage profile rather than adsorption energy alone. In addition, the Fe–B framework remains largely preserved under high alkali-metal loading, indicating good structural compatibility with substantial adsorption. Overall, FeB_3_ provides a favorable balance among capacity, ion mobility, voltage characteristics, and structural integrity, highlighting the potential of boron-rich metalloborophenes as high-performance anode materials for alkali-metal-ion batteries.

## Figures and Tables

**Table 1:** Average adsorption energies (*E*_ads_^avg^, eV) of Li, Na, and K atoms on the FeB_3_ monolayer at different adsorption numbers *n* in a 3 × 3 supercell.

	Number of Adsorbed Atoms, *n*									
Adsorbate	4	6	12	14	18	20	24	28	32	36
Li	−1.58	−1.54	−1.46	−1.43	−1.39	−1.33	−1.15	−0.99	−0.78	−0.55
Na	−1.56	−1.45	−1.27	−1.23	−1.14	−1.04	−0.88	−0.64	—	—
K	−1.91	−1.75	−1.34	−1.23	−0.97	—	—	—	—	—

**Note:** *n* represents the number of adsorbed alkali-metal atoms in the 3 × 3 FeB_3_ supercell. The values are average adsorption energies in eV. The dash (—) indicates that the corresponding adsorption configuration was not retained as an energetically favorable stable state.

**Table 2:** Summary of maximum adsorption loadings and electrochemically accessible storage capacities of Li, Na, and K on the FeB_3_ monolayer.

Parameter	Li	Na	K
Highest loading investigated (atoms)	36	36	36
Maximum stable adsorption loading (atoms)	36	28	18
Positive-OCV storage limit (atoms)	28	24	18
Accessible fraction (%)	77.8	85.7	100.0
Maximum adsorption capacity (mAh g^−1^)	1214.0	944.2	606.9
Electrochemically accessible capacity (mAh g^−1^)	944.2	809.3	606.9

## Data Availability

The original contributions presented in this study are included in the article/supplementary material. Further inquiries can be directed to the corresponding author.

## Supplementary Materials

The following supporting information can be downloaded at: https://www.mdpi.com/article/10.3390/ma19214493/s1. Supplementary Information provides additional details supporting the main text, including the side-view atomic structures and ELF maps of pristine and fully alkali-metal-adsorbed FeB_3_ monolayers (Figure S1), the representative adsorption sites and corresponding adsorption energies of individual Li, Na, and K atoms on the FeB_3_ surface (Figure S2 and Table S1), and the optimized stable adsorption configurations at different coverages for Li, Na, and K storage (Figures S3–S5).
