# Supplementary material for: Planar Hypercoordinate FeB3 Monolayer for Universal Alkali-Ion Storage with Ultrahigh Capacity and Ultralow Diffusion Barriers
Source: Materials (Basel). 2026 Oct 25;19(21):4493. doi: 10.3390/ma19214493 (PMC13764654; doi:10.3390/ma19214493)
Supplement: Supplementary file 1 [file materials-19-04493-s001.zip › materials-4622979-supplementary.pdf]

materials

Supplementary Material

# Planar Hypercoordinate $FeB_3$ Monolayer for Universal Alkali-Ion Storage with Ultrahigh Capacity and Ultralow Diffusion Barriers

**Huanhuan Xie [1,2,*], Kehan Zhang [1], Dong Liang [1], Pinpin Jing [1] and Changding Guo [3]**

[1] Key Laboratory of Magnetic Molecules and Magnetic Information Materials of the Ministry of Education, School of Materials Science and Engineering, Shanxi Normal University, Taiyuan 030031, China; 225111085@sxnu.edu.cn (K.Z.); 226125059@sxnu.edu.cn (D.L.); 226125036@sxnu.edu.cn (P.J.)
[2] Center for Integrated Nanostructure Physics (CINAP), Sungkyunkwan University, Suwon 16419, Republic of Korea
[3] Department of Energy Science, Sungkyunkwan University, Suwon 16419, Republic of Korea; cd.guo@skku.edu
* Correspondence: 20210090@sxnu.edu.cn

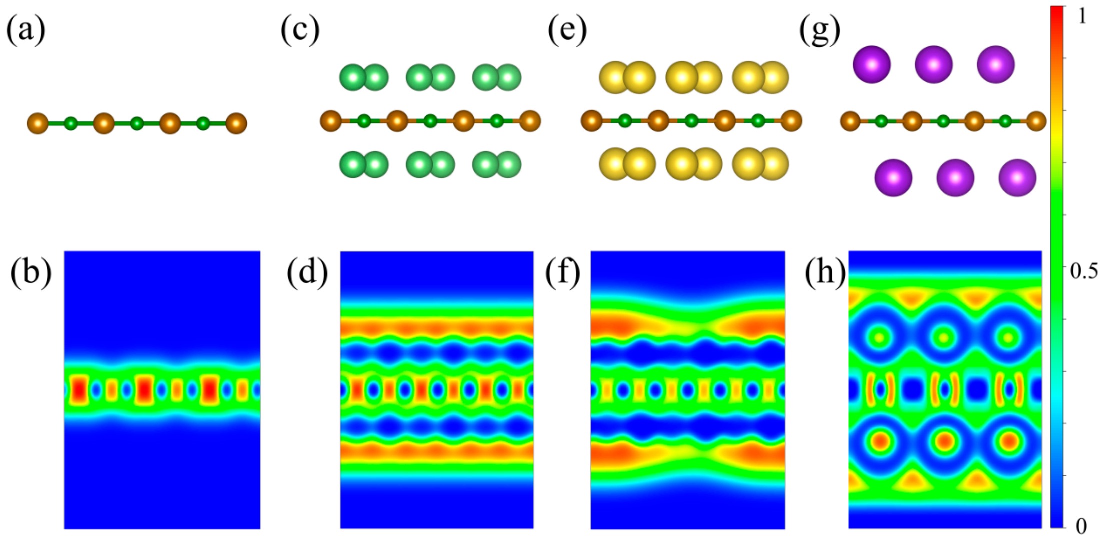


**Figure S1.** Side-view atomic structures and corresponding electron localization function (ELF) maps of pristine and alkali-metal-adsorbed $FeB_3$ monolayers. (a, b) Pristine $FeB_3$ monolayer. (c, d) Li-adsorbed $FeB_3$ monolayer with 36 Li atoms. (e, f) Na-adsorbed $FeB_3$ monolayer with 28 Na atoms. (g, h) K-adsorbed $FeB_3$ monolayer with 18 K atoms. Brown, green, light green, yellow, and purple spheres represent Fe, B, Li, Na, and K atoms, respectively. The ELF color scale ranges from 0 (blue) to 1 (red).

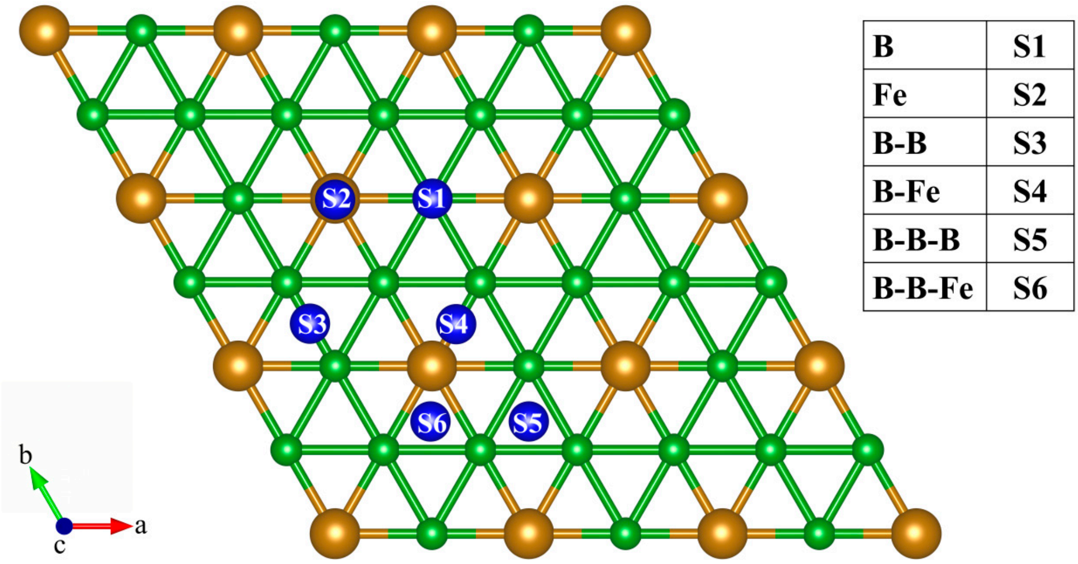


**Figure S2.** Schematic illustration of the representative adsorption sites considered on the $FeB_3$ monolayer: the top site above a B atom (S1), the top site above an Fe atom (S2), the bridge site above a B-B bond (S3), the bridge site above a B-Fe bond (S4), the B-B-B hollow site (S5), and the B-B-Fe hollow site (S6). The blue spheres mark the examined adsorption sites.

**Table S1.** Adsorption energies of a single Li, Na, or K atom at the considered adsorption sites on the $FeB_3$ monolayer.

| Adsorbate | Adsorption energy (eV) | | | | | |
|---|---|---|---|---|---|---|
| | S1 (B top) | S2 (Fe top) | S3 (B–B bridge) | S4 (B–Fe bridge) | S5 (B–B–B hollow) | S6 (B–B–Fe hollow) |
| Li | −1.61 | −1.46 | −1.66 | −1.60 | −1.68 | −1.54 |
| Na | −1.77 | −1.65 | −1.78 | −1.76 | −1.79 | −1.71 |
| K | −2.27 | −2.16 | −2.29 | −2.28 | −2.30 | −2.17 |

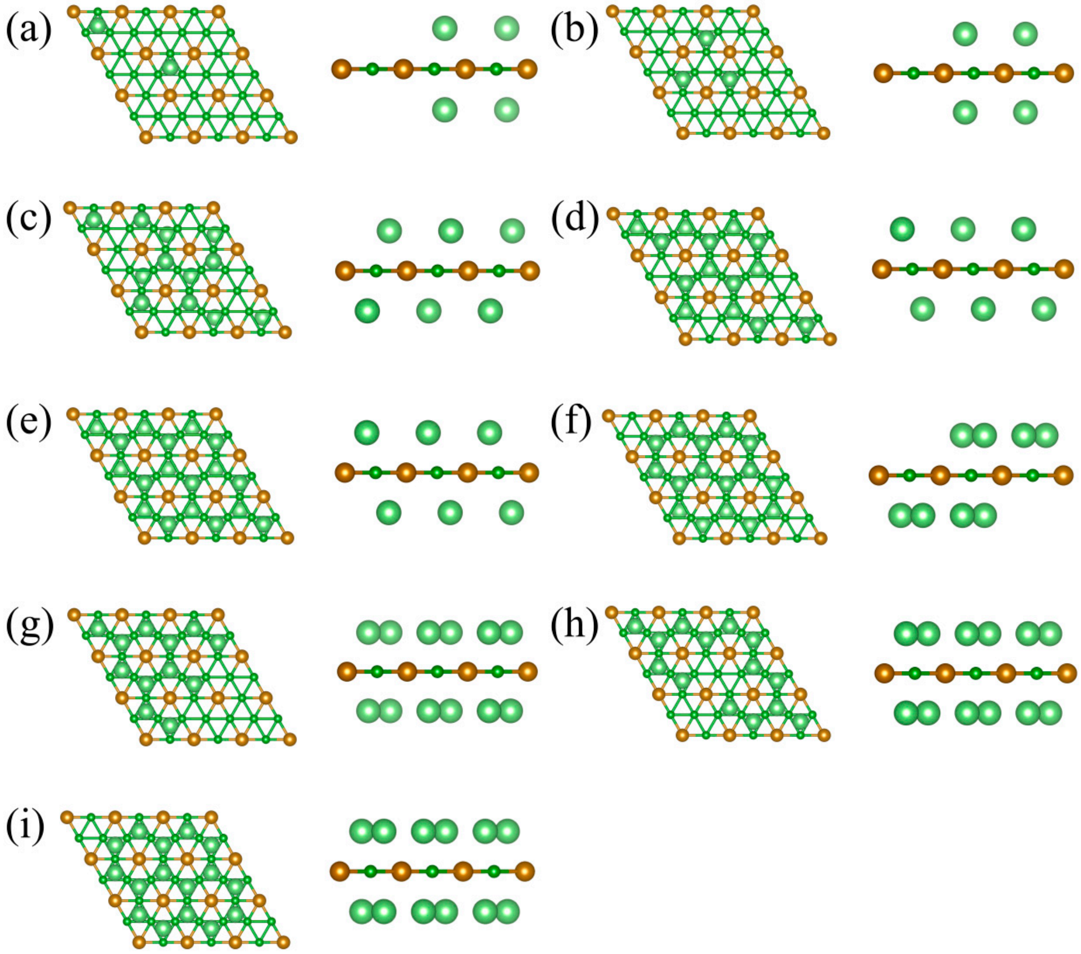


**Figure S3.** Optimized most stable adsorption configurations of Li atoms on the $FeB_3$ monolayer at coverages of n = 2, 4, 6, 8, 14, 18, 24, 32, and 36, corresponding to panels (a)–(i). For each panel, the left image shows the top view and the right image shows the side view; configurations are arranged sequentially from left to right according to increasing adsorption number. Light green, brown, and green spheres represent Li, B, and Fe atoms, respectively.

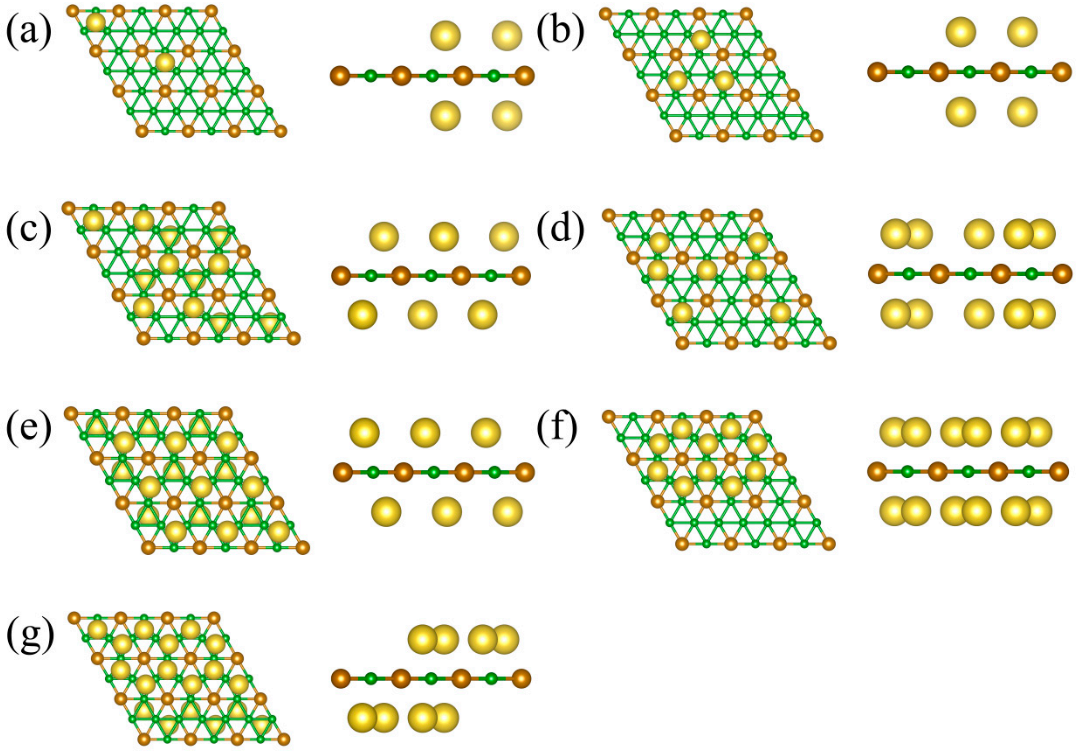


**Figure S4.** Optimized most stable adsorption configurations of Na atoms on the $FeB_3$ monolayer at coverages of n = 4, 6, 12, 14, 18, 20, and 24, corresponding to panels (a)–(g). For each panel, the left image shows the top view and the right image shows the side view; configurations are arranged sequentially from left to right according to increasing adsorption number. Yellow, brown, and green spheres represent Na, B, and Fe atoms, respectively.

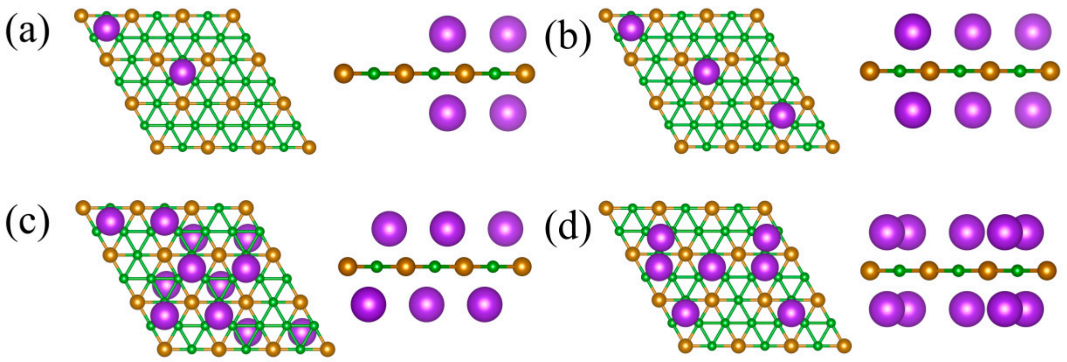


**Figure S5.** Optimized most stable adsorption configurations of K atoms on the $FeB_3$ monolayer at coverages of n = 4, 6, 12, and 14 corresponding to panels (a)–(d). For each panel, the left image shows the top view and the right image shows the side view; configurations are arranged sequentially from left to right according to increasing adsorption number. Purple, brown, and green spheres represent K, B, and Fe atoms, respectively.
